# High-Value Bioactive Molecules Extracted from Microalgae

**DOI:** 10.3390/microorganisms13092018

**Published:** 2025-08-29

**Authors:** Carla Arenas Colarte, Iván Balic, Óscar Díaz, Adrián A. Moreno, Maximiliano J. Amenabar, Tamara Bruna Larenas, Nelson Caro Fuentes

**Affiliations:** 1 Centro de Investigación Austral Biotech, Facultad de Ciencias, Universidad Santo Tomás, Santiago 8320000, Chile; carenas8@santotomas.cl (C.A.C.); tbruna@santotomas.cl (T.B.L.); 2 Área Prioritaria de Investigación (API3), Programa Fitogen, Departamento de Acuicultura y Recursos Agroalimentarios, Universidad de Los Lagos, Osorno 5311157, Chile; ivan.balic@ulagos.cl (I.B.); oscar.diaz@ulagos.cl (Ó.D.); 3 Centro de Biotecnología Vegetal, Facultad de Ciencias de la Vida, Universidad Andres Bello, Santiago 8370146, Chile; adrian.moreno@unab.cl; 4 Escuela de Biotecnología, Facultad de Ciencias, Universidad Santo Tomás, Santiago 8320000, Chile; mamenabar@santotomas.cl

**Keywords:** microalgae, sustainability, bioactive compounds, extraction, microbial, plant

## Abstract

Microalgae are unicellular photosynthetic organisms with considerable genetic diversity and remarkable metabolic capacity, positioning them as sustainable cellular biorefineries. They can be cultivated in open or closed systems, influenced by physical and chemical variables such as light, temperature, and nutrient availability. These conditions modulate the synthesis of valuable biomolecules, including proteins, lipids, polysaccharides, and secondary metabolites. Microalgae are especially notable for their high protein content (up to 70% *w*/*w* in *Spirulina* sp.), polyunsaturated fatty acids (e.g., DHA and EPA), and β-glucans with bioactive properties. Choosing the correct extraction method (mechanical, enzymatic or combined) is very important to obtain and preserve the functionality of these compounds. Despite their biotechnological potential in functional foods, pharmaceuticals, and biofuels, industrial development faces challenges such as extraction efficiency, scalability, and regulatory approval. This review compiles current knowledge on the nutritional and bioactive potential of microalgae, highlights advances in extraction technologies and discusses their potential applications in health-oriented industrial innovation.

## 1. Introduction

Microalgae are photosynthetic microscopic organisms that exhibit remarkable genetic diversity and a highly efficient photosynthetic apparatus, capable of transforming sunlight and carbon dioxide into valuable compounds. Their cultivation is influenced by the light intensity received and the type of substrate used in the production system, with the aim of optimizing growth rates and biomass generation [1]. Microalgae are characterized by their high biodiversity, with an estimated 800,000 species, although only 40,000 to 50,000 have been described. These microorganisms have been classified into the following phyla: *Chlorophyta*, *Bacillariophyta*, *Cyanobacteria*, *Ochrophyta*, *Haptophyta*, *Rhodophyta*, *Euglenozoa*, *Charophyta*, *Miozoa*, and *Prasinodermatophyta*. Despite this high diversity, only a limited number of microalgal species have been explored for biotechnological applications. For example, in Europe, only 18 species of *Chlorophyta* and 10 species of the *Bacillariophyta phylum* have been used [2]. Culture conditions for microalgal biomass production can be open culture systems, which are more cost-effective but prone to contamination, or closed culture systems, which are more expensive and require sterile environments (photobioreactors). Although open culture systems are cost-effective due to their simple design and easy access to resources, they have disadvantages due to culture contamination, space requirements, and difficulty in controlling the process [3]. The generation of microalgal biomass is faster than that of plants, resulting in greater raw material yields with simpler extraction processes and an abundance of biomolecules for diverse applications. Potential applications include wastewater treatment, carbon dioxide sequestration, human and animal nutrition, active ingredients for the cosmetics industry, the production of high-value compounds, natural colorants, stable isotope generation, biofertilizers, and the development of pharmaceutical products [4]. These microorganisms are known as light-activated cellular factories that produce bioactive compounds consisting of primary metabolites (such as lipids, proteins, and carbohydrates) and secondary metabolites (such as pigments, polyphenols, and vitamins). These molecules are characterized by their bioactivity, meaning they exert a biological effect on a target with health benefits such as disease suppression. Humans and other higher organisms are unable to produce most of these molecules, or can only do so in minimal quantities, so their external incorporation through the diet is recommended to prevent deficiencies that could be harmful [5]. The health-promoting properties of microalgae are due to the accumulation of bioactive molecules extracted from the cellular biomass or the utilization of the entire biomass, depending on the objective. The production of these molecules is specific to each species and depends on the cultivation conditions [6]. These molecules must meet the following requirements for a target product to be valuable: they must accumulate at high concentrations within microalgal cells grown under standard conditions, or be generated in large quantities as an adaptive response to non-ideal culture conditions (such as nutrient availability, or physicochemical factors such as pH, temperature, and lighting), or when cells are exposed to chemical and/or physical stress, which induces increased production of the desired molecules [7]. Microalgae are an excellent option to contribute to the growing demand for natural, healthier, and more sustainable products that fortify foods, strengthen the immune system without the use of pharmaceuticals, and replace synthetic antibiotics with new, naturally derived compounds that possess equal or greater efficacy against antibiotic-resistant pathogens. Compounds extracted from microalgae can fill nutritional gaps in foods and are a good alternative for vegetarian and vegan consumers who do not consume animal products. However, despite the widespread commercial availability of algae-derived bioactive compounds with proven efficacy, social perceptions regarding their health benefits remain somewhat uncertain [8]. The algae-based products market is currently valued at $4.7 billion and is projected to reach $6.4 billion by 2026, at a compound annual growth rate of 6.3%. Asia, the United States, and Oceania lead the production of these products, while Europe accounts for only 5% of the market [9]. Microalgae biomass production is considered low-cost due to the use of natural resources (water, sunlight, carbon dioxide) for its cultivation, which are inexpensive and readily available. Several species, such as *Chlorella*, *Dunaliella*, and *Haematococcus*, are currently marketed as functional foods due to their ability to store bioactive and nutritional molecules [10]. However, the constraints on the progress of industrial microalgae biotechnology are primarily due to the costs associated with the subsequent biomass cultivation and biorefinery processes, which account for up to 50–80% of the total. Due to these constraints, research must focus on improving cultivation techniques, discovering and analyzing new biomolecules, developing more efficient extraction methods, and designing processes that require less energy [8].

## 2. Types of Bioactive Compounds: High-Value Primary Metabolites

**Figure 1:**
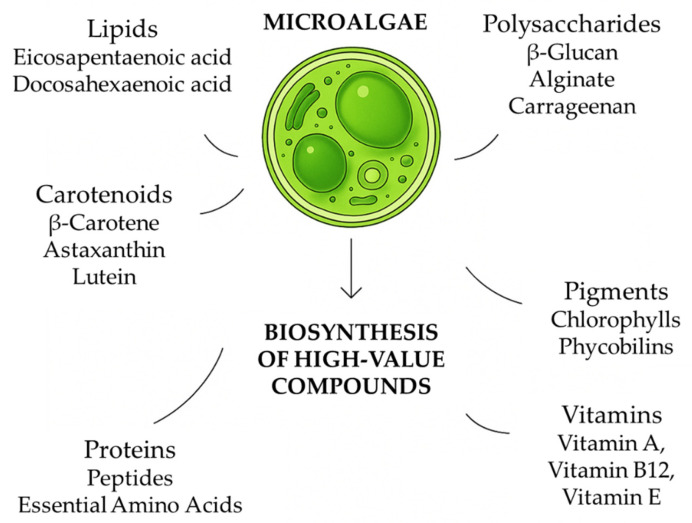
Biosynthesis of high-value compounds by microalgae. Microalgae convert sunlight, carbon dioxide, and nutrients into a wide range of bioactive compounds through metabolic pathways regulated by environmental and culture conditions. These include primary metabolites such as proteins, lipids (including polyunsaturated fatty acids like DHA and EPA), and carbohydrates, as well as secondary metabolites like pigments (e.g., β-carotene, astaxanthin), polyphenols, and vitamins.

Bioactive molecules are chemical substances present in foods consumed by humans. These molecules have the ability to modulate the metabolic processes of organisms that acquire them in their diet. High-value bioactive substances are a large group of compounds (carotenoids, polyphenols, polysaccharides, peptides, vitamins, etc.) with different chemical structures (hydrophilic or lipophilic), found at varying of concentrations both in food and in the human body, specific to each species, widely distributed in nature, with different mechanisms of action and effectiveness against free radicals. The bioactive compounds predominantly present in microalgae include carotenoids (such as β-carotene and astaxanthin), polyunsaturated fatty acids (PUFAs) such as docosahexaenoic acid (DHA) and eicosapentaenoic acid (EPA), as well as polysaccharides such as β-glucan (Figure 1). In recent years, biotechnology applied to microalgae has gained increasing importance. Bioactive molecules extracted from microalgae have a wide range of applications, ranging from biomass production for human and animal feed to the development of environmental solutions and pharmaceutical research [11].

**Figure 2:**
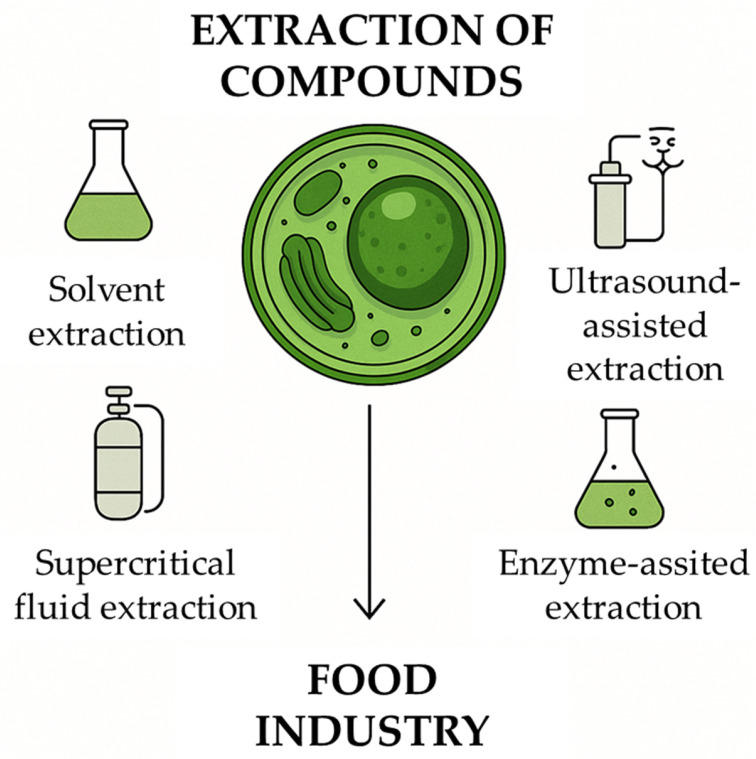
Various methods are employed to recover bioactive molecules from microalgal biomass, each with distinct advantages depending on the target compounds and desired purity. The figure illustrates solvent extraction, ultrasound-assisted extraction, supercritical fluid extraction, and enzyme-assisted extraction. These techniques enhance the release and preservation of valuable compounds such as lipids, pigments, polysaccharides, and polyphenols, contributing to the efficiency and sustainability of downstream processing in food, pharmaceutical, and cosmetic industries.

Figure 1 represents the main metabolites of interest biosynthesized by microalgae. These metabolites have high biotechnological value due to their nutritional, pharmacological, and functional properties, making microalgae a promising source for applications in the food, cosmetics, nutraceutical, and agricultural industries. The diversity and quantity of these compounds can vary depending on the microalgal species, environmental conditions, and cultivation methods. Microalgae have the capacity to synthesize this wide range of metabolites of industrial interest, which are obtained through conventional and emerging extraction techniques (Figure 2), including solvent extraction, ultrasound-assisted extraction, supercritical fluid extraction, and enzyme-assisted extraction. These metabolites are subsequently used in the production of various industrial applications.

### 2.1. Proteins

Proteins are essential nutrients for human nutrition, and most come from animal sources. Currently, the rapid increase in the world’s population has generated protein shortages in several countries. This has led to a need to increase the production of protein-rich foods, primarily in areas with limited agricultural resources, and to promote innovation in the search for non-animal proteins. Microalgae have emerged as a promising source of protein, constituting a sustainable alternative to conventional animal and plant proteins. These microorganisms stand out for their high protein content as well as their nutritional, functional, and bioactive properties, making them suitable for use in food, feed, and nutraceutical products. The remarkable diversity of microalgae species, estimated at approximately 200,000, offers vast potential for the identification of new proteins with distinct nutritional profiles and potential beneficial effects on human health [12]. Microalgal biomass offers a dietary protein source with enormous potential. Its protein content rivals that of traditional foods such as fish, soy, and eggs in quantity and quality. For example, *Chlorella* sp. and *Spirulina* sp. have up to 70% protein content, depending on the variety. In fact, these species lead the global demand for microalgae [13]. Proteins extracted from microalgae, peptides such as Leu-Asn-Gly-Asp-Val-Trp and some essential amino acids provide powerful health benefits as they are essential for cells to optimally perform their functions [14]. Functionally, microalgal proteins exhibit emulsifying, water-retaining, and gel-forming properties, enabling applications in bakery, dairy, and meat analogues [15]. A study revealed that the amount of essential amino acids in certain microalgae is comparable to, and even higher in some cases than, that of eggs (raw and cooked) and soybeans (cooked), and the WHO/FAO reference values. Specifically, *Nannochloropsis* sp., *Phaeodactylum tricornutum*, *Scenedesmus obliquus* and *Arthrospira platensis* showed higher levels of histidine, isoleucine, leucine, lysine, threonine, and valine than the reference values [16]. As specific protein fragments, bioactive peptides are essential for the biological functioning of most organisms. These peptides have the potential to transform sectors such as the medical, cosmetic, and food industries due to their high safety, efficacy, selectivity, and easy digestion, and research in this field is booming [17]. For this reason, microalgae-derived proteins and peptides represent a sustainable source with the potential to replace animal products and contribute to preventing and reducing the incidence and impact of diet-related diseases, such as non-alcoholic fatty liver disease and inflammatory bowel diseases [18]. This potential is largely attributed to the composition of microalgal biomass, which is made up of several components, among which proteins are the most abundant in most species. It also contains other substances, many of which are biologically active. To access and utilize this valuable biomolecule, particularly protein, efficient and species-specific extraction methods are required. The protein extraction method must be tailored to each type of microalgae, as they are composed of tough cell walls, which makes them difficult to break and requires more appropriate techniques [19]. Microalgae proteins are encased in complex cell wall structure, making the extraction process one of the most important challenges to be solved. Therefore, for this process to be efficient, extraction techniques are essential for successful recovery. This stage is key to ensuring the nutritional and functional quality of the proteins, as the goal is to simplify their application in the food industry. Extraction methods generally include biomass harvesting and drying, as well as more specific processing techniques to recover and purify protein extracts. The choice of the appropriate technique depends on the microalgae strain, the type of protein, and the desired application, as the goal is to avoid the decrease or loss of the protein’s biological function. Figure 2 illustrates some of the most used extraction techniques, including solvent extraction, ultrasound-assisted extraction, enzyme-assisted extraction, and supercritical fluid extraction, highlighting their relevance in preserving compound integrity and maximizing yields.

In recent years, significant efforts have been directed toward refining extraction techniques to improve efficiency and selectivity. The most recent advances in the extraction of these bioactive molecules focus on improving methods that prevent damage and maintain high yields [15]. Table 1 summarizes and compares traditional and combined methods for extracting proteins from microalgae.

### 2.2. Lipids

A critical challenge in using microalgae for renewable fuel and chemical production is the effective extraction of their lipids, composed primarily of triglycerides and free fatty acids (collectively known as FA oils), from microalgae [30]. Microalgal oil is commercially attractive due to its short reproductive cycle, high lipid-storage capacity, and, consequently, its abundance of saturated, monounsaturated, and long-chain polyunsaturated fatty acids. Polyunsaturated fatty acids from microalgae typically account for a high percentage of the total fatty acid content, primarily eicosapentaenoic acid (EPA) and docosahexaenoic acid (DHA). These acids contain more than two double bonds in their structure and depending on the position of the first unsaturated bond, they are divided into ω3, ω6, ω7, and ω9, with ω3 and ω6 being the most important for regulating biological processes in the organism [17]. Microalgae are also capable of producing different types of lipids (glycolipids, phospholipids, and triacylglycerides), which are composed of fatty acids with 12 to 24 carbon atoms. These lipids play fundamental roles in cellular metabolism, acting as energy stores, energy donors, structural components of membranes, and mediators in processes such as intracellular signaling, modulation of gene expression, cell–cell communication, exocytosis, and vesicular trafficking. The lipid content in microalgae can reach up to 50% of their dry weight [31]. Lipid production in microalgae can be regulated by changing culture conditions such as light, carbon dioxide concentration, temperature, and nutrient availability. Controlling and modifying these parameters can increase biomass production and, consequently, the accumulation of lipids of interest [32]. Microalgae, regardless of their variations, represent a significant source of PUFAs, including EPA and DHA, two essential fatty acids with high nutritional value [33]. The microalgae that primarily produce these lipids are *Haematococcus*, *Spirulina*, *Schizochytrium* and *Crypthecodinium* [34]. Algae grown under stress conditions produce a large amount of lipids. In fact, microalgae are an excellent source of several biofuels (biobutane, biodiesel, bioethanol, biohydrogen, and biomethane) [35]. Extracting the maximum amount of polyunsaturated fatty acids (PUFAs) from microalgae demands both efficient extraction and effective purification. Since a comprehensive isolation process is lacking, current extraction techniques are undergoing continuous adaptation [36]. Table 2 summarizes and compares conventional, modern and combined methods for obtaining lipids from microalgae.

In this context, the production of PUFAs from microalgae presents significant opportunities for progress but also poses challenges that must be addressed. A decline in biomass production costs, along with strengthened regulatory frameworks, collaboration between academia and industry, access to venture capital financing, and technological advances, have been key factors driving development in this field. The high costs of industrial-scale microalgae production remain a challenge, primarily due to the low proportion of PUFAs in the biomass.

### 2.3. Polysaccharides

Polysaccharides (PS) are high-molecular-weight macromolecules found in all living organisms. These molecules exhibit structural and functional diversity at the biochemical level. They can be classified as homopolysaccharides, formed by the repetition of a single type of monosaccharide, or as heteropolysaccharides, consisting of two or more different types of sugars. Furthermore, their structure can be linear or branched, and they can contain various substituent groups in their main chain [47]. Photosynthetic microorganisms are classified into three groups based on their location within the cell and function: structural photosynthesis in cell walls, intracellular storage photosynthesis, and extracellular photosynthesis. Many microalgae generate and release a gelatinous matrix of polysaccharides, called mucilage, which likely serves as a protective barrier against environmental changes [48]. In microalgae, polysaccharides serve as structural, protective, and energy-storage compounds. Their utilization is primarily concentrated in species belonging to the genera *Rhodella*, *Chlorella*, *Porphyridium*, *Isochrysis*, *Tetraselmis*, and *Phaeodactylum* [49]. Microalgal polysaccharides (PS) have been shown to possess unique rheological properties, positioning them as potential novel gelling and thickening agents. Although the market is currently dominated by PS from seaweed and bacteria, the unusual characteristics of microalgal PS, along with the expected reduction in their production costs soon, could facilitate their incorporation into new applications. Furthermore, numerous studies have demonstrated diverse biological activities associated with these compounds, including antitumor, anticoagulant, antiparasitic, antioxidant, antibacterial, anti-inflammatory, and immunomodulatory properties. All these qualities make microalgae a promising source for the industrial valorization of these polysaccharides [50]. Microalgal polysaccharides have great potential in food science and technology, as they offer nutritionally viable alternatives. They are also widely used in biomaterials, such as alginate and chitosan, two of the most versatile biodegradable polymers available [51]. Unlike other food sources, microalgae prebiotics are innovative functional foods. They are resistant to digestion, allowing them to promote the growth of beneficial bacteria (probiotics) in the gut [52]. Microalgae also represent a relevant source of prebiotics, which include various native or modified forms of polysaccharides, such as xylooligosaccharides, galactooligosaccharides, alginate oligosaccharides, neoagarooligosaccharides, galactans, arabinoxylans and β-glucans [53]. Microalgae polysaccharides are considered safe, characterized by their stability and versatility. Their composition includes sugars such as glucose, galactose, and xylose; they also typically contain β-glucans, cellulose, hemicellulose, uronic acids, and fucose [54]. Another polysaccharide produced by microalgae is beta-1,3-glucans, which, depending on the species, are also known as mycolaminarin, chrysolaminarin, or laminarin. These are involved in carbon storage and form key componentes of the cell wall structures of microorganisms [55]. The specific structure of β-glucan depends on the source, and variations in structure can affect solubility and biological activity. The microalga *Euglena graciliz*, for example, can accumulate large amounts of β-1,3-glucan intracellularly, which can represent more than 90% of the cell’s dry weight [5]. The linear structure of beta-glucans boosts their biological activity, improving their ability to modulate the immune response and act as antioxidants. These polysaccharides are stored as granules inside the cell, making processing easier, lowering costs, and preserving the molecule’s functionality [56]. The significant commercial and scientific interest in these compounds stems from their diverse biological activities, including immunomodulation and antioxidant properties, and their potential applications in functional foods and medicines. Microalgal β-glucans are especially noteworthy due to their unique structure, low production cost, and scalability [57]. After microalgae have grown under conditions suitable for PS synthesis, methods must be implemented to recover the molecules. It is essential to find a balance between polymer purity and the cost of extraction and purification processes. Polysaccharides soluble in the culture medium are the easiest to recover [58]. Table 3 summarizes and compares conventional, modern and combined methods for obtaining polysaccharides from microalgae.

Emerging methods for extracting polysaccharides from microalgae represent more sustainable and environmentally friendly alternatives. However, industrial-scale research is still limited, so additional studies are needed to optimize these processes and reduce their energy demand. To promote their application in human health, future research should focus on improving culture conditions, refining chemical characterization techniques, and optimizing extraction procedures for bioactive compounds. In this context, as reported in Table 3, several innovative technologies with strong potential to address current limitations are being evaluated.

## 3. Types of Bioactive Compounds: High-Value Secondary Metabolites of Microalgae

In addition to containing significant amounts of primary metabolites (such as proteins, carbohydrates, and polyunsaturated fatty acids), the health benefits of microalgae are mainly attributed to their content of high-value secondary metabolites. These secondary metabolites are non-nutrient-containing compounds produced by plants as a defense mechanism against environmental stressors [68]. Microalgae are a rich source of pigments, such as chlorophylls, carotenoids, and flavonoids. Typical pigment producing species include *Coelastrella striolata*, *Haematococcus pluvialis*, *Spirulina platensis*, *Dunaliella salina*, *Nanochloropsis* sp., *Chlorella* sp., and others [69]. Table 4 summarizes the most important metabolites obtained from microalgae, along with their respective applications.

Obtaining carotenoids from microalgae involves several stages: The process typically begins with harvesting the culture, then drying the biomass, breaking down the cell walls using mechanical methods, and finally performing solvent-based extraction and purification [105]. Carotenoids extraction often requires a large number of reagents, so when selecting the most appropriate extraction method, one must consider both the chemical structure of the target carotenoid and the microalgae species used. At the laboratory level, conventional methods using nonpolar solvents are commonly employed due to their simplicity and ease of application. However, for carotenoids such as lutein and β-carotene, biphasic systems, which combine different solvents, have been shown to yield higher extraction efficiencies [106]. There are also more sophisticated extraction methods available, such as ultrasound-assisted extraction, pressurized fluid extraction, and subcritical and supercritical solvent extraction. These are more environmentally friendly alternatives to organic solvents. One notable example is supercritical carbon dioxide extraction: a fast, safe, cost-effective, and efficient method for recovering carotenoids [79]. Careful selection of the extraction method is essential to maximize recovery and preserve the functionality of secondary metabolites such as carotenoids and phenolic compounds in microalgae. A summary and comparison of the main extraction strategies—encompassing both conventional approaches and emerging technologies, along with their characteristics, advantages, and reported applications—are presented below (Table 5).

## 4. Perspectives

Currently, the production of compounds from microalgal biomass has been the subject of numerous studies, gaining relevance due to its potential application in the development of sustainable and environmentally friendly technologies. However, a large proportion of these studies involve the cultivation of microalgae in synthetic media and under controlled laboratory conditions. The data obtained reflects a strong dependence of the metabolite profile on the strain used and the stress conditions imposed (such as light intensity, temperature, pH, and nutrient concentration, among other factors). Extraction and purification remain a challenge due to the diverse structures of the target compounds and the physiological variability among microalgae species. To advance in this area, it is proposed that future research adopt a systematic approach to biomolecule recovery, structured in four key stages: (1) appropriate selection of microalgal strains, (2) standardization of culture conditions, (3) detailed characterization of the biomolecules produced, and (4) development of efficient extraction and functionalization processes.

In particular, the development of simpler and more cost-efficient extraction protocols is considered a priority. In this regard, efforts should focus on establishing methodologies that enable the direct use of raw biomass or require minimal pre-treatment. Emerging technologies such as enzyme-assisted extraction, ultrasound, and the use of supercritical fluids are emerging as promising tools to enhance their application on an industrial scale. The integrated use of microalgae represents a promising strategy to address global challenges related to nutrition, energy, and the environment. In this context, the commercialization of microalgae-based products has grown significantly in recent years, driven by increasing consumer interest in sustainable and nutritious alternatives. Nonetheless, the industrial use of microalgae remains fragmented and lacks standardization, which limits broader market integration. Despite growing commercial interest, their application remains largely confined to high-value niche markets, likely due to low production yields and high processing costs. However, progress in scaling up cultivation systems, optimizing biomass processing, and implementing integrated biorefinery approaches, along with a better understanding of the factors influencing the production of bioactive compounds, could enhance the value chain soon.

## 5. Conclusions

The valorization of microalgae as a source of biomolecules represents a viable and sustainable alternative to address current challenges in the energy, environmental, and food sectors. While significant progress has been made in the cultivation and processing of microalgal biomass, limitations persist related to the standardization of cultivation conditions, variability in metabolite production, and the complexity of extraction and purification processes. For this reason, a system must be implemented that encompasses everything from the correct selection of strains to the extraction method to ensure the functionality of the obtained compounds and the economic viability of these processes. Combining extraction methods (mechanical and enzymatic) has been reported to achieve greater process efficiency, purer extracts, and lower energy expenditure. To fully harness the potential of microalgae as a sustainable biotechnology platform, it will be essential to promote interdisciplinary research and develop policies that support their large-scale production. In conclusion, to expand the application of microalgae in diverse industrial sectors, the use of emerging technologies, optimizing cultivation resources, and integrating biomass must be encouraged.

## Figures and Tables

**Table 1:** Comparison of protein extraction methods from microalgae.

Extraction Methods	Advantages	Disadvantages	Efficiency/Observations	References
Mechanical (microsphere grinding, high-pressure homogenization)	High efficiency of cell disruption	It requires expensive equipment and high energy consumption	Milling extracted >90% of the total protein in *Chlorella vulgaris*	[20,21,22,23,24]
Enzymatic (proteases, carbohydrases)	High selectivity, preserves functionality	High cost of enzymes	At *Scenedesmus obliquus*, 27% multi-enzyme extraction; 21% cellulase extraction	[25,26]
Combined (lyophilization, micronization + enzymes)	Synergy increases performance	Greater complexity of the process	Triples the concentration of bioactive peptides in *Chlorella vulgaris*	[27,28]
Ultrasound	Improve efficiency when combined with other methods	Detailed individual efficiency is not reported	Produces cellular disruption by cavitation	[29]

**Table 2:** Comparison of lipid extraction methods from microalgae.

Extraction Methods	Advantages	Disadvantages	Efficiency/Observations	References
Conventional (Soxhlet, Folch, Bligh-Dyer)	Simple and economical	Use of toxic solvents and prolonged periods	Not environmentally recommended; low sustainability	[37,38,39]
Modern (SFE, UAE, MAE)	Higher performance and lower environmental impact	MAE not suitable for thermo-sensitive compounds	UAE and MAE induce cell rupture by cavitation or pressure	[40,41]
Enzymatic (cellulase, pectinase, etc.)	High specificity, mild conditions	Requires precise operating conditions	Cellulase doubled the extraction in *Nannochloropsis* sp.	[42,43,44,45]
Combined (enzymes + mechanical or chemical methods)	Increases overall efficiency	Needs specific optimization	Extraction > 80% in *Chlorella vulgaris* with combined method	[38,45,46]

**Table 3:** Comparison of polysaccharide extraction methods from microalgae.

Extraction Methods	Advantages	Disadvantages	Observations	References
Conventional (hot water, acid or alkaline extraction)	Profitable, easy to apply	Risk of co-extraction of proteins or other compounds	Highly soluble in water and alcohols	[58,59]
Assisted (ultrasound, microwave)	Increase performance and reduce energy and time	May require special equipment	RSM + microwaves improve biological activity and reduces consumption	[60]
Enzymatic (lysozymes, cellulase, chitinases)	High efficiency, low energy consumption	It depends on the composition of the cell wall	Strain-specific enzymes; ideal cellulase for *Nannochloropsis* sp.	[61,62,63,64]
Supercritical (CO_2_)	Green alternative without toxic solvents	Limited research on an industrial scale	Promising for sustainable production	[65,66]
Combined/Genetic Technologies	Optimizes performance and metabolic understanding	In development, requires further research	Genetic engineering applied to *Chlamydomonas* to improve performance	[67]

**Table 4:** Summary of secondary metabolites of microalgae.

Metabolite	Description/Properties	Microalgae Producers	Applications	References
Chlorophyll	Photosynthetic green pigment; types b, c, d, f depending on the species; 0.5–1% of dry weight	*Chlorella vulgaris*, *Scenedesmus dimorphus*, *Pavlova lutheri*, *Chlamydomonas reinhardtii*, *Monoraphidium dybowskii*	Natural coloring (food, cosmetics, toothpaste); antioxidant supplement (chlorophyllin)	[70,71,72,73]
Carotenoids	Tetraterpenoid pigments; high antioxidant bioactivity; intensified under stress	*Coelastrella striolata*, *Haematococcus pluvialis*, *Spirulina platensis*, *Dunaliella salina*, *Nanochloropsis* sp., *Chlorella* sp.	Animal feed, pharmaceuticals, cosmetics and nutraceuticals	[73,74,75,76,77,78,79]
β- carotene	Provitamin A; yellow-orange pigment; high Dunaliella salina content (98.5%)	*Dunaliella salina*	Nutritional supplements, cosmetics, food antioxidants	[80,81,82,83]
Astaxanthin	Red pigment; accumulates under stress; more potent antioxidant than vitamin E	*Haematococcus pluvialis*, *Chlorella zofingiensis*, *Chlorococcum* sp.	Nutraceuticals, aquaculture, cosmetics, antioxidant supplements	[84,85,86,87]
Lutein	Filters blue light (~500 nm); lipophilic antioxidant; 0.4–0.6% dry weight in *Muriellopsis* sp.	*Muriellopsis* sp., *Scenedesmus almeriensis*, *Chlorella protothecoides*	Eye health, food additive, natural coloring	[88,89]
Fucoxanthin	Orange pigment; antioxidant, anti-inflammatory, anticancer properties	*Tisochrysis lutea*, *Phaeodactylum tricornutum*, *Odontella aurita*, *Navicula* sp.	Cosmetics, functional foods, poultry farming, aquaculture	[90,91,92,93]
Zeaxanthin	Protects the macula; antioxidant and neuroprotective	*Chromochloris zofingiensis*, *Dunaliella salina*	Eye health, nutraceuticals, natural supplements	[94,95]
Phenolic compounds	They include phenolic acids, flavonoids, tannins, and stilbenes. Antioxidant activity depends on functional structure.	*Scenedesmus* sp., *Dunaliella salina*, *Chlorella minutissima*	Pharmaceutical, cosmetics and food industry	[96,97,98,99,100,101,102]
Vitamins (A, C, E, B12)	Antioxidant activity: synthesis and accumulation depend on species and conditions	*Isochrysis galbana*, *Euglena gracilis*, *Skeletonema marinei*, *Tetraselmis suecica*, *Chlorella* sp.	Human health, vegan diets, cosmetics, supplements	[103,104]

**Table 5:** Extraction methods of carotenoids and compounds phenolics from microalgae.

Type of Compound	Extraction Methods	Characteristics of the Method	Observations/Examples	References
Carotenoids	Conventional with nonpolar solvents	Simple, easy to apply in the laboratory	Suitable for general carotenoids; low sustainability	[96]
	Two-phase solvent systems	Greater efficiency in specific carotenoids such as lutein and β-carotene	Optimize performance by combining solvents of different polarity	[97]
	Ultrasound, pressurized fluids, sub/supercritical solvents	Ecological and efficient alternatives	Supercritical CO_2_: a fast, safe, and efficient process	[79]
Compounds Phenolics	Solvent extraction (water, ethanol, methanol)	Varies depending on species, cultivation phase and solvent used	Ethanol/water in *Scenedesmus* sp. increased phenols and quercetin; aqueous extract of *D. salina* showed high levels in the stationary phase	[106]
	HPLC for identification	Detects individual phenolic compounds	Quercetin, gallic acid, chlorogenic acid and 4-hydroxybenzoic acid identified	[107]

## Data Availability

The original contributions presented in this study are included in the article. Further inquiries can be directed to the corresponding author.

## Abbreviations

The following abbreviations are used in this manuscript:

DHA	Docosahexaenoic acid
EPA	Eicosapentaenoic acid
FA	Fatty acid
MAE	Microwave-assisted extraction
PS	Polysaccharides
PUFA	Polyunsaturated fatty acid
SFE	Supercritical fluid extraction

## References

[1] Patel A.K., Joun J.M., Hong M.E. (2019). Effect of light conditions on mixotrophic cultivation of green microalgae. Bioresour. Technol..

[2] Fernandes T., Cordeiro N. (2022). Microalgae as Sustainable Biofactories to Produce High-Value Lipids: Biodiversity, Exploitation, and Biotechnological Applications. Mar. Drugs.

[3] Eze C.N., Aoyagi H., Ogbonna J.C. (2020). Simultaneous accumulation of lipid and carotenoid in freshwater green microalgae *Desmodesmus subspicatus* LC172266 by nutrient replete strategy under mixotrophic condition. Korean J. Chem. Eng..

[4] Occhipinti P.S., Russo N., Foti P., Zingale I.M., Pino A., Romeo F.V., Randazzo C.L., Caggia C. (2023). Current challenges of microalgae applications: Exploiting the potential of non-conventional microalgae species. J. Sci. Food Agric..

[5] Barsanti L., Gualtieri P. (2022). Algae: Anatomy, Biochemistry, and Biotechnology.

[6] Eze C.N., Ogbonna I.O., Aoyagi H., Ogbonna J.C. (2021). Comparison of growth, protein and carotenoid contents of some freshwater microalgae and effects of urea and cultivation in a photobioreactor with reflective broth circulation guide on *Desmodesmus subspicatus* LC172266. Braz. J. Chem. Eng..

[7] Miguel S.P., Ribeiro M.P., Otero A., Coutinho P. (2021). Application of microalgae and microalgal bioactive compounds in skin regeneration. Algal Res..

[8] Barsanti L., Gualtieri P. (2023). Glucans, Paramylon and Other Algae Bioactive Molecules. Int. J. Mol. Sci..

[9] Blockchain Market Worth $67.4 Billion by 2026—Report by MarketsandMarkets™. https://www.marketsandmarkets.com/PressReleases/algae-product.asp.

[10] Sathasivam R., Radhakrishnan R., Hashem A. (2019). Microalgae metabolites: A rich source for food and medicine. Saudi J. Biol. Sci..

[11] Hassan S., Meenatchi R., Pachillu K., Bansal S., Brindangnanam P., Arockiaraj J., Kiran G.S., Selvin J. (2023). Identification and characterization of the novel bioactive compounds from microalgae and cyanobacteria for pharmaceutical and nutraceutical applications. J. Basic Microbiol..

[12] Silva S.C., Almeida T., Colucci G., Santamaria-Echart A., Manrique Y.A., Dias M.M., Barros L., Fernandes A., Colla E., Barreiro M.F. (2022). Spirulina (*Arthrospira platensis*) protein-rich extract as a natural emulsifier for oil-in-water emulsions: Optimization through a sequential experimental design strategy. Colloids Surf. A Physicochem. Eng. Asp..

[13] Acquah C., Ekezie F.G., Udenigwe C.C. (2021). Cultured Microalgae for the Food Industry: Current and Potential Applications.

[14] Kumar R., Hegde A.S., Sharma K., Parmar P., Srivatsan V. (2022). Microalgae as a sustainable source of edible proteins and bioactive peptides—Current trends and future prospects. Food Res. Int..

[15] García-Encinas J.P., Ruiz-Cruz S., Juárez J., Ornelas-Paz J.d.J., Del Toro-Sánchez C.L., Márquez-Ríos E. (2025). Proteins from Microalgae: Nutritional, Functional and Bioactive Properties. Foods.

[16] Siahbalaei R., Kavoosi G., Noroozi M. (2021). Protein nutritional quality, amino acid profile, anti-amylase and anti-glucosidase properties of microalgae: Inhibition and mechanisms of action through in vitro and in silico studies. LWT—Food Sci. Technol..

[17] Wu J., Gu X., Yang D., Xu S., Wang S., Chen X., Wang Z. (2021). Bioactive substances and potentiality of marine microalgae. Food Sci. Nutr..

[18] Eilam Y., Khattib H., Pintel N., Avni D. (2023). Microalgae-Sustainable Source for Alternative Proteins and Functional Ingredients Promoting Gut and Liver Health. Glob. Chall..

[19] Menaa F., Wijesinghe U., Thiripuranathar G., Althobaiti N.A., Albalawi A.E., Khan B.A., Menaa B. (2021). Marine Algae-Derived Bioactive Compounds: A New Wave of Nanodrugs. Mar. Drugs.

[20] Cunha S.A., Coscueta E.R., Nova P., Silva J.L., Pintado M.M. (2022). Bioactive Hydrolysates from *Chlorella vulgaris*: Optimal Process and Bioactive Properties. Molecules.

[21] Callejo-López J.A., Ramírez M., Bolívar J., Cantero D. (2019). Main variables affecting a chemical-enzymatic method to obtain protein and amino acids from resistant microalgae. J. Chem..

[22] Rahman M.M., Hosano N., Hosano H. (2022). Recovering microalgal bioresources: A review of cell disruption methods and extraction technologies. Molecules.

[23] Safi C., Frances C., Ursu A.V., Laroche C., Pouzet C., Vaca-Garcia C., Pontalier P.Y. (2015). Understanding the effect of cell disruption methods on the diffusion of *Chlorella vulgaris* proteins and pigments in the aqueous phase. Algal Res..

[24] Postma P.R., Miron T.L., Olivieri G., Barbosa M.J., Wijffels R.H., Eppink M.H.M. (2015). Mild disintegration of the green microalgae *Chlorella vulgaris* using bead milling. Bioresour. Technol..

[25] Günerken E., D’Hondt E., Eppink M.H., Garcia-Gonzalez L., Elst K., Wijffels R.H. (2015). Cell disruption for microalgae biorefineries. Biotechnol. Adv..

[26] Amiri M., Hosseini S.E., Asadi G., Khayambashi B., Abedinia A. (2024). Optimization of microalgae protein extraction from *Scenedesmus obliquus* and investigating its functional properties. LWT—Food Sci. Technol..

[27] Zhang R., Chen J., Mao X., Qi P., Zhang X. (2019). Anti-inflammatory and anti-aging evaluation of pigment-protein complex extracted from *Chlorella pyrenoidosa*. Mar. Drugs.

[28] Mendes Costa M., Pinheiro Spínola M., Diogo Alves V., Mestre Prates J.A. (2024). Improving protein extraction and peptide production from *Chlorella vulgaris* using combined mechanical/physical and enzymatic pre-treatments. Heliyon.

[29] Martínez-Sanz M., Garrido-Fernández A., Mijlkovic A., Krona A., Martínez-Abad A., Coll-Marqués J.M., López-Rubio A., Lopez-Sanchez P. (2020). Composition and rheological properties of microalgae suspensions: Impact of ultrasound processing. Algal Res..

[30] Menegazzo M.L., Fonseca G.G. (2019). Biomass recovery and lipid leaching processes for microalgae biofuels production: A review. Renew. Sustain. Energy Rev..

[31] Dolganyuk V., Belova D., Babich O., Prosekov A., Ivanova S., Katserov D., Patyukov N., Sukhikh S. (2020). Microalgae: A Promising Source of Valuable Bioproducts. Biomolecules.

[32] Leal E., de Beyer L., O’Connor W., Dove M., Ralph P.J., Pernice M. (2021). Production optimisation of *Tisochrysis lutea* as a live feed for juvenile Sydney rock oysters, *Saccostrea glomerata*, using large-scale photobioreactors. Aquaculture.

[33] Nascimento T.C., Cazarin C.B.B., Maróstica M.R., Mercadante A.Z., Jacob-Lopes E., Zepka L.Q. (2020). Microalgae carotenoids intake: Influence on cholesterol levels, lipid peroxidation and antioxidant enzymes. Food Res. Int..

[34] Barkia I., Saari N., Manning S.R. (2019). Manning, microalgae for high-value products towards human health and nutrition. Mar. Drugs.

[35] Arun J., Gopinath K.P., SundarRajan P., Felix V., JoselynMonica M., Malolan R. (2020). A conceptual review on microalgae biorefinery through thermochemical and biological pathways: Bio-circular approach on carbon capture and wastewater treatment. Bioresour. Technol. Rep..

[36] Li X.P., Liu J.P., Chen G.Y., Zhang J.G., Wang C.B., Liu B. (2019). Extraction and purification of eicosapentaenoic acid and docosahexaenoic acid from microalgae: A critical review. Algal Res. Biomass Biofuels Bioprod..

[37] Sharma A.K., Chintala V., Ghodke P., Prasher P., Patel A. (2020). Extraction and purification of PUFA from microbial biomass. Nutraceutical Fatty Acids from Oleaginous Microalgae.

[38] Zhou J., Wang M., Saraiva J.A., Martins A.P., Pinto C.A., Prieto M.A., Simal-Gandara J., Cao H., Xiao J., Barba F.J. (2022). Extraction of lipids from microalgae using classical and innovative approaches. Food Chem..

[39] Jesus S.D., Ferreira G.F., Moreira L.S., Regina M., Maciel W., Maciel R. (2019). Comparison of several methods for effective lipid extraction from wet microalgae using green solvents. Renew. Energy.

[40] Nagappan S., Devendran S., Tsai P.C., Dinakaran S., Dahms H.U., Ponnusamy V.K. (2019). Passive cell disruption lipid extraction methods of microalgae for biofuel production—A review. Fuel.

[41] Sallet D., Souza P.O., Fischer L.T., Ugalde G., Zabot G.L., Mazutti M.A., Kuhn R.C. (2019). Ultrasound-assisted extraction of lipids from *Mortierella isabellina*. J. Food Eng..

[42] Zhang Y., Kong X., Wang Z., Sun Y., Zhu S., Li L., Lv P. (2018). Optimization of enzymatic hydrolysis for effective lipid extraction from microalgae *Scenedesmus* sp.. Renew. Energy.

[43] He Y., Zhang B., Guo S., Guo Z., Chen B., Wang M. (2020). Sustainable biodiesel production from the green microalga Nannochloropsis: Novel integrated processes from cultivation to enzyme-assisted extraction and lipid ethanolysis. Energy Convers. Manag..

[44] Alavijeh R.S., Karimi K., Wijffels R.H., van den Berg C., Eppink M. (2020). Combined bead milling and enzymatic hydrolysis for efficient fractionation of lipids, proteins, and carbohydrates of *Chlorella vulgaris* microalgae. Bioresour. Technol..

[45] Sierra L.S., Dixon C.K., Wilken L.R. (2017). Enzymatic cell disruption of the microalgae *Chlamydomonas reinhardtii* for lipid and protein extraction. Algal Res..

[46] Santin A., Russo M.T., Ferrante M.I., Balzano S., Orefice I., Sardo A. (2021). Highly Valuable Polyunsaturated Fatty Acids from Microalgae: Strategies to Improve Their Yields and Their Potential Exploitation in Aquaculture. Molecules.

[47] Delattre C., Pierre G., Laroche C., Michaud P. (2016). Production, extraction and characterization of microalgal and cyanobacterial exopolysaccharides. Biotechnol. Adv..

[48] Shnyukova E.I., Zolotareva E.K. (2017). Ecological role of exopolysaccharides of *Bacillariophyta*: A review. Algologia.

[49] Chandrarathna H.P.S.U., Liyanage T.D., Edirisinghe S.L., Dananjaya S.H.S., Thulshan E.H.T., Nikapitiya C., Oh C., Kang D.H., De Zoysa M. (2020). Marine Microalgae, *Spirulina* maxima-Derived Modified Pectin and Modified Pectin Nanoparticles Modulate the Gut Microbiota and Trigger Immune Responses in Mice. Mar. Drugs.

[50] Gaignard C., Gargouch N., Dubessay P., Delattre C., Pierre G., Laroche C., Fendri I., Abdelkafi S., Michaud P. (2019). New horizons in culture and valorization of red microalgae. Biotechnol. Adv..

[51] Mahcene Z., Khelil A., Hasni S., Akman P.K., Bozkurt F., Birech K. (2020). Development and characterization of sodium alginate based active edible films incorporated with essential oils of some medicinal plants. Int. J. Biol. Macromol..

[52] Patel A.K., Singhania R.R., Awasthi M.K., Varjani S., Bhatia S.K., Tsai M.L. (2021). Emerging prospects of macro- and microalgae as prebiotic. Microb. Cell Fact..

[53] Gouda M., Tadda M.A., Zhao Y., Farmanullah F., Chu B., Li X., He Y. (2022). Microalgae Bioactive Carbohydrates as a Novel Sustainable and Eco-Friendly Source of Prebiotics: Emerging Health Functionality and Recent Technologies for Extraction and Detection. Front. Nutr..

[54] Costa J.A.V., Lucas B.F., Alvarenga A.G.P., Moreira J.B., de Morais M.G. (2021). Microalgae Polysaccharides: An Overview of Production, Characterization, and Potential Applications. Polysaccharides.

[55] Ma M., Li Y., Chen J., Wang F., Yuan L., Li Y., Zhang B., Ye D., Han D., Jin H. (2021). High-Cell-Density Cultivation of the Flagellate Alga Poterioochromonas Malhamensis for Biomanufacturing the Water-Soluble β-1,3-Glucan with Multiple Biological Activities. Bioresour. Technol..

[56] Gao L., Zhao X., Liu M., Zhao X. (2022). Characterization and antibacterial activities of carboxymethylated paramylon from *Euglena gracilis*. Polymers.

[57] Kumar V., Bhoyar M.S., Mohanty C.S., Chauhan P.S., Toppo K., Ratha S.K. (2025). Untapping the potential of algae for β-glucan production: A review of biological properties, strategies for enhanced production and future perspectives. Carbohydr. Polym..

[58] Laroche C. (2022). Exopolysaccharides from Microalgae and Cyanobacteria: Diversity of Strains, Production Strategies, and Applications. Mar. Drugs..

[59] Liu F., Chen H., Qin L., Al-Haimi A.A.N.M., Xu J., Zhou W., Zhu S., Wang Z. (2023). Effect and characterization of polysaccharides extracted from *Chlorella* sp. by hot-water and alkali extraction methods. Algal Res..

[60] Zhang N., Chen W., Li X., Chen X., Wang Y., Huang G., Wang J., Jia Z. (2024). Enzyme-assisted ultrasonic extraction and antioxidant activities of polysaccharides from *Schizochytrium limacinum* meal. Foods.

[61] Malvis A., Morales J.J.P., Klose L., Liese A. (2023). Enzyme-assisted extraction of Ulvan from the green macroalgae *Ulva fenestrata*. Molecules.

[62] Sanjeewa K.K.A., Herath K.H.I.N.M., Kim Y.S., Jeon Y.J., Kim S.K. (2023). Enzyme-Assisted Extraction of Bioactive Compounds from Seaweeds and Microalgae. TrAC Trends Anal. Chem..

[63] Peng H., Xv X., Cui X., Fu Y., Zhang S., Wang G., Chen X., Song W. (2023). Physicochemical Characterization and Antioxidant Activity of Polysaccharides from *Chlorella* sp. by Microwave-Assisted Enzymatic Extraction. Front. Bioeng. Biotechnol..

[64] Gurpilhares D.d.B., Cinelli L.P., Simas N.K., Pessoa A., Sette L.D. (2019). Marine Prebiotics: Polysaccharides and Oligosaccharides Obtained by Using Microbial Enzymes. Food Chem..

[65] Tzima S., Georgiopoulou I., Louli V., Magoulas K. (2023). Recent advances in supercritical CO_2_ extraction of pigments, lipids and bioactive compounds from microalgae. Molecules.

[66] Nguyen D.T., Johir M.A.H., Mahlia T.M.I., Silitonga A.S., Zhang X., Liu Q., Nghiem L.D. (2024). Microalgae-derived biolubricants: Challenges and opportunities. Sci. Total Environ..

[67] Masi A., Leonelli F., Scognamiglio V., Gasperuzzo G., Antonacci A., Terzidis M.A. (2023). *Chlamydomonas reinhardtii:* A Factory of Nutraceutical and Food Supplements for Human Health. Molecules.

[68] Ampofo J.O., Ngadi M. (2020). Elicitación fenólica asistida por ultrasonidos y potencial antioxidante de los brotes de frijol común (*Phaseolus vulgaris*). Ultrasonido. Sonochem..

[69] Hamidi M., Kozani P.S., Kozani P.S., Pierre G., Michaud P., Delattre C. (2019). Marine Bacteria versus Microalgae: Who Is the Best for Biotechnological Production of Bioactive Compounds with Antioxidant Properties and Other Biological Applications?. Mar. Drugs.

[70] Sun H., Wang Y., He Y., Liu B., Mou H., Chen F., Yang S. (2023). Microalgae-Derived Pigments for the Food Industry. Mar. Drugs.

[71] Duppeti H., Chakraborty S., Das B.S., Mallick N., Kotamreddy J. (2017). Rapid assessment of algal biomass and pigment contents using diffuse reflectance spectroscopy and chemometrics. Algal Res..

[72] Faraloni C., Torzillo G., Cvetkovic D., Nikolic G. (2017). Synthesis of Antioxidant Carotenoids in Microlagae in Response to Physiological Stress. Carotenoids.

[73] Ngamwonglumlert L., Devahastin S., Chiewchan N. (2017). Molecular structure, stability and cytotoxicity of natural green colorants produced from *Centella asiatica* L. leaves treated by steaming and metal complexations. Food Chem..

[74] Liu C., Hu B., Cheng Y., Guo Y., Yao W., Qian H. (2021). Carotenoids from Fungi and Microalgae: A Review on Their Recent Production, Extraction, and Developments. Bioresour. Technol..

[75] Zheng H., Wang Y., Li S., Wu Q., Feng X., Zheng Y., Leong Y.K., Lee D.-J., Chang J.-S. (2022). Lutein production by microalgae using corn starch wastewater pretreated with rapid enzymatic hydrolysis. Bioresour. Technol..

[76] Patel A.K., Albarico F., Perumal P.K., Vadrale A.P., Nian C.T., Chau H.T., Anwar C., Wani H., Pal A., Saini R. (2022). Algae as an emerging source of bioactive pigments. Bioresour. Technol..

[77] Zaytseva A., Chekanov K., Zaytsev P., Bakhareva D., Gorelova O., Kochkin D., Lobakova E. (2021). Sunscreen Effect Exerted by Secondary Carotenoids and Mycosporine-like Amino Acids in the Aeroterrestrial Chlorophyte *Coelastrella rubescens* under High Light and UV-A Irradiation. Plants.

[78] Maoka T. (2020). Carotenoids as natural functional pigments. J. Nat. Med..

[79] Ambati R.R., Gogisetty D., Aswathanarayana R.G., Ravi S., Bikkina P.N., Bo L., Yuepeng S. (2019). Industrial potential of carotenoid pigments from microalgae: Current trends and future prospects. Crit. Rev. Food Sci. Nutr..

[80] Rajput A., Singh D.P., Khattar J.S., Swatch G.K., Singh Y. (2022). Evaluation of growth and carotenoid production by a green microalgae *Scenedesmus quadricauda* PUMCC 4.1. 40. under optimized culture conditions. J. Basic Microbiol..

[81] Nwoba E.G., Rohani T., Raeisossadati M., Vadiveloo A., Bahri P.A., Moheimani N.R. (2021). Monochromatic light filters to enhance biomass and carotenoid productivities of *Dunaliella salina* in raceway ponds. Bioresour. Technol..

[82] Rammuni M.M., Ariyadasa T.U., Nimarshana P.H.V., Attalage R.A. (2019). Comparative Assessment on the Extraction of Carotenoids from Microalgal Sources: Astaxanthin from *H. pluvialis* and β-carotene from *D. salina*. Food Chem..

[83] Cezare-Gomes E.A., Mejia-da-Silva L.D., Perez-Mora L.S., Matsudo M.C., Ferreira-Camargo L.S., Singh A.K., de Carvalho J.C.M. (2019). Potential of Microalgae Carotenoids for Industrial Application. Appl. Biochem. Biotechnol..

[84] Sun H., Li X., Ren Y., Zhang H., Mao X., Lao Y., Wang X., Chen F. (2020). Boost carbon availability and value in algal cell for economic deployment of biomass. Bioresour. Technol..

[85] Mularczyk M., Michalak I., Marycz K. (2020). Astaxanthin and Other Nutrients from *Haematococcus pluvialis*—Multifunctional Applications. Mar. Drugs.

[86] Lu Q., Li H., Zou Y., Liu H., Yang L. (2021). Astaxanthin as a microalgal metabolite for aquaculture: A review on the synthetic mechanisms, production techniques, and practical application. Algal Res..

[87] Berman J., Zorrilla-López U., Farré G., Zhu C., Sandmann G., Twyman R.M., Capell T., Christou P. (2015). Nutritionally important carotenoids as consumer products. Phytochem. Rev..

[88] Andrade D., Colozzi-Filho A., Guedes C., Lima F., Machineski G., Matos M., Andrade D.S., Colozzi-Filho A. (2014). Main products of algal biomass and their biotechnological applications. Microalgae from Continental Waters: Potentials and Challenges of Cultivation.

[89] D’Alessandro E.B., Antoniosi Filho N.R. (2016). Concepts and studies on lipid and pigments of microalgae: A review. Renew. Sustain. Energy Rev..

[90] Leong Y.K., Chen C.-Y., Varjani S., Chang J.-S. (2022). Producing fucoxanthin from algae—Recent advances in cultivation strategies and downstream processing. Bioresour. Technol..

[91] Li Y., Sun H., Wu T., Fu Y., He Y., Mao X., Chen F. (2019). Storage carbon metabolism of *Isochrysis zhangjiangensis* under different light intensities and its application for co-production of fucoxanthin and stearidonic acid. Bioresour. Technol..

[92] Mohamadnia S., Tavakoli O., Faramarzi M.A. (2021). Enhancing production of fucoxanthin by the optimization of culture media of the microalga *Tisochrysis lutea*. Aquaculture.

[93] Sun H., Yang S., Zhao W., Kong Q., Zhu C., Fu X., Zhang F., Liu Z., Zhan Y., Mou H. (2022). Fucoxanthin from marine microalgae: A promising bioactive compound for industrial production and food application. Crit. Rev. Food Sci. Nutr..

[94] Ren Y., Sun H., Deng J., Huang J., Chen F. (2021). Carotenoid Production from Microalgae: Biosynthesis, Salinity Responses and Novel Biotechnologies. Mar. Drugs.

[95] Idenyi J.N., Eya J.C., Nwankwegu A.S., Nwoba E.G. (2022). Aquaculture sustainability through alternative dietary ingredients: Microalgal value-added products. Eng. Microbiol..

[96] Wang J., Hu X., Chen J., Wang T., Huang X., Chen G. (2022). The Extraction of β-Carotene from Microalgae for Testing Their Health Benefits. Foods.

[97] Kalra R., Gaur S., Goel M. (2021). Microalgae bioremediation: A perspective towards wastewater treatment along with industrial carotenoids production. J. Water Proc. Eng..

[98] Cichoński J., Chrzanowski G. (2022). Microalgae as a Source of Valuable Phenolic Compounds and Carotenoids. Molecules.

[99] Albuquerque B.R., Heleno S.A., Oliveira M.B.P.P., Barros L., Ferreira I.C.F.R. (2021). Phenolic Compounds: Current Industrial Applications, Limitations and Future Challenges. Food Funct..

[100] Jimenez-Lopez C., Pereira A.G., Lourenço-Lopes C., Garcia-Oliveira P., Cassani L., Fraga-Corral M., Prieto M.A., Simal-Gandara J. (2021). Main Bioactive Phenolic Compounds in Marine Algae and Their Mechanisms of Action Supporting Potential Health Benefits. Food Chem..

[101] Bulut O., Akın D., Sönmez Ç., Öktem A., Yücel M., Öktem H.A. (2019). Phenolic Compounds, Carotenoids, and Antioxidant Capacities of a Thermo-Tolerant *Scenedesmus* sp. (Chlorophyta) Extracted with Different Solvents. J. Appl. Phycol..

[102] Andriopoulos V., Gkioni M.D., Koutra E., Mastropetros S.G., Lamari F.N., Hatziantoniou S., Kornaros M. (2022). Total Phenolic Content, Biomass Composition, and Antioxidant Activity of Selected Marine Microalgal Species with Potential as Aquaculture Feed. Antioxidants.

[103] Sansone C., Brunet C. (2019). Promises and Challenges of Microalgal Antioxidant Production. Antioxidants.

[104] Del Mondo A., Smerilli A., Sané E., Sansone C., Brunet C. (2020). Challenging Microalgal Vitamins for Human Health. Microb. Cell Factories.

[105] Smerilli A., Orefice I., Corato F., Ruban A., Brunet C. (2017). Photoprotective and antioxidant responses to light spectrum and intensity variations in the coastal diatom Skeletonema marinoi. Environ. Microbiol..

[106] Fawcett C.A., Senhorinho G.N.A., Laamanen C.A., Scott J.A. (2022). Microalgae as an Alternative to Oil Crops for Edible Oils and Animal Feed. Algal Res..

[107] Santiago-Morales I.S., Trujillo-Valle L., Márquez-Rocha F.J., López Hernández J.F. (2018). Tocopherols, Phycocyanin and Superoxide Dismutase from Microalgae as Potential Food Antioxidants. Appl. Food Biotechnol..

